# Detection of vision and /or hearing loss using the interRAI Community Health Assessment aligns well with common behavioral vision/hearing measurements

**DOI:** 10.1371/journal.pone.0223123

**Published:** 2019-10-03

**Authors:** Andrea Urqueta Alfaro, Dawn M. Guthrie, Natalie A. Phillips, M. Kathleen Pichora-Fuller, Paul Mick, Cathy McGraw, Walter Wittich

**Affiliations:** 1 School of Optometry, University of Montréal, Montréal, Quebec, Canada; 2 Centre de Recherche Interdisciplinaire en Réadaptation du Montréal Métropolitain, Montréal, Quebec, Canada; 3 Department of Kinesiology & Physical Education, Wilfrid Laurier University, Waterloo, Ontario, Canada; 4 Department of Health Sciences, Wilfrid Laurier University, Waterloo, Ontario, Canada; 5 Department of Psychology, Concordia University, Montréal, Quebec, Canada; 6 Department of Psychology, University of Toronto, Mississauga, Ontario, Canada; 7 Department of Surgery, College of Medicine, University of Saskatchewan, Saskatoon, Saskatchewan, Canada; 8 CRIR/Lethbridge-Layton-Mackay Rehabilitation Centre of West-Central Montreal,Montréal, Quebec, Canada; 9 CRIR/Institut Nazareth et Louis-Braille du CISSS de la Montérégie-Centre, Montréal, Quebec, Canada; Radboud University, NETHERLANDS

## Abstract

This study’s main objective was to assess the sensitivity and specificity of the interRAI Community Health Assessment (CHA) for detecting the presence of vision loss (VL), hearing loss (HL) or both (Dual Sensory Loss, DSL) when compared against performance-based measures of vision and hearing. The interRAI CHA and the Montreal Cognitive Assessment (MoCA) were administered to 200 adults (61+ years of age) who had VL, HL or DSL. We calculated the sensitivity and specificity of the interRAI CHA for detecting sensory impairments using as the gold standard performance based measurements of hearing (pure-tone audiogram) and vision (distance acuity) as determined from the rehabilitation centre record. Results were divided according to participants’ cognitive status, as measured by the MoCA and the Cognitive Performance Scale (CPS, embedded within the interRAI CHA). Overall, sensitivity was 100% for VL, 97.1% for HL, and 96.9% for DSL. Specificity was at least 93% in all three groups. In participants who failed the MoCA (i.e., at risk of mild cognitive impairment), the sensitivity was 100% for VL, 96.8% for HL and 96.2% for DSL; in those who were not at risk, the sensitivity was 100% for VL, and 97.4% for HL and DSL. In participants classified by the CPS as borderline intact or mild cognitively impaired, sensitivity was 100% in all groups; in those classified as cognitively intact, sensitivity was 100% for VL, 97.0% for HL, and 96.8% for DSL. These results suggest that the interRAI CHA detects VL, HL, and DSL in high agreement with performance-based measurements of vision and hearing. The interRAI CHA shows high accuracy even in participants with mild cognitive difficulties. Since results were found in a specific population of older rehabilitation clients who all had sensory difficulties, further research is needed to understand its role in screening in other more diverse groups.

## Background

Dual sensory loss (DSL) refers to the combination of concurrent vision and hearing loss irrespective of age or the order of onset of the sensory losses [1]. DSL constitutes a unique disability in which a person may not be able to compensate for the loss in one of these two senses by using the other sense [2,3]. DSL negatively affects a person’s ability to communicate, acquire information, perform daily activities, and fully participate in social environments [4,5]. Persons with DSL are at greater risk for many health issues (e.g., impaired mobility, depression) [5], and compared to persons with other disabilities, are more likely to have a low socio-economic status, and have poorer educational outcomes [6].

The incidence of DSL is higher in older adults (65+ years of age) with prevalence estimates varying across studies depending on their methodologies and the sub-groups of older adults included in their samples. Across four countries (Canada, US, Finland and Belgium), the prevalence of DSL in older adults ranges between 9.7% and 33.9% in long-term care facilities, and between 13.4% and 24.6% in home care [7].

Given the myriad of negative effects associated to DSL in older adults, the detection and evaluation of DSL is of utmost importance [8]. Once diagnosed, persons with DSL can access rehabilitation services for sensory loss that can help to alleviate the negative consequences of DSL on a person’s health and functioning. Additionally, medical professionals must be aware of a person’s sensory abilities in order to communicate information in ways that are accessible to these individuals [9]. Although the incidence of DSL is higher in older adults, many first-line health care providers (i.e., the first ones to encounter a client in need of assessment) operate under the basic assumption that their clients can hear and see them. In reality, given the relationship between aging and sensory impairment, it is estimated that as many as one in three individuals over 50 years of age have either reduced vision, impaired hearing, or DSL [1,8].

Medical doctors diagnose DSL using performance based measurements of hearing (pure-tone audiogram) and vision (distance acuity). First-line health care providers such as social workers do not typically administer performance based assessments and instead identify DSL based on their clinical observation and client’s self-report. The interRAI Community Health Assessment (CHA), and its Deafblind Supplement (DbS) is currently the only standardized interview instrument for adults (18 years of age or older) that helps first-line health care and social services providers to identify the needs, strengths and challenges for someone with DSL [10,11]. Furthermore, this assessment is widely used by Ontario agencies that provide community support services. It is necessary to investigate the accuracy with which the interRAI CHA detects older adults with DSL. This information will help evaluate the appropriateness of using this assessment and of possibly expanding its use beyond Ontario. This research is particularly urgent because as the populations of most developed countries are aging, the prevalence of DSL is expected to increase. Indeed, the number of people who are 85 years of age or older is increasing faster than any other age group in the population, and these individuals are the most likely to experience the challenges associated with DSL [12,13].

The interRAI CHA is an assessment of overall health and functional abilities that guides an assessor in terms of developing a service plan [11]. It consists of roughly 150 items, and four supplemental assessments, one of which is the DbS. Responses indicating problems on the two items on the CHA that refer to the person’s functional vision and hearing “trigger” an identification of the person as likely having DSL such that a more comprehensive assessment with the DbS is warranted. The DbS includes an additional 150 items that probe details about the person’s vision and hearing and other issues relevant for an individual with DSL (e.g., age of onset of sensory loss, diagnoses, communication ability, psychosocial well-being) [14]. Based on the information gathered, the assessor then decides how to proceed in terms of referrals (e.g., referral to programs/resources specific to persons with DSL) and implementation of a service plan. Research shows that the interRAI CHA and DbS, and associated scales, have good internal consistency and convergent validity [15,16], as well as some preliminary evidence of acceptable inter-rater reliability [17]. This assessment was created by interRAI (http://www.interrai.org/), a not-for-profit research network of 100 members from 35 countries, who have a mandate to develop and test assessment systems that aim to improve the quality of life and delivery of services for vulnerable populations, including older persons and those with disabilities. The interRAI instruments are based on input from content experts, clinicians and service providers, are used internationally, including being mandated in several regions in Canada and are supported by studies evaluating their psychometric properties [18–20].

The detection of DSL in older adults using the interRAI CHA partly depends on the respondent’s capacity to understand and respond to questions regarding functional vision and hearing. Such capacity may be limited in older adults due to the changes in cognition associated with age that range from cognitive decline to cognitive impairment including dementia [21]. Mild cognitive impairment (MCI) constitutes an intermediate clinical state between normal cognitive aging and dementia that is characterized by impairment in one or more cognitive domains that exceeds what is considered normal for a persons’ age and education; and preservation of independence in functional activities which negates the diagnosis of very mild dementia [22]. Several clinical MCI subtypes have been proposed depending on whether there is significant memory impairment and the number of impaired cognitive domains [23]. Prevalence of MCI in adults older than 65 years ranges between 3% and 19%, and more than half of persons diagnosed with MCI convert to dementia within 5 years [24]. It is anticipated that within the next two decades, cognitive impairment including dementia will be among the top 4 burdens of disease in middle- and high-income countries [25].

Moreover, the combined presence of vision, hearing and cognitive impairment is associated with greater levels of communication difficulties (i.e., understanding others and being understood by others), compared to cognitive impairment alone [5]. For those who receive home care, compared to older adults without DSL (including those with single sensory impairment), those with DSL are more likely to have moderate/severe cognitive impairment. For those living in long-term care facilities, those with DSL are more likely than those without DSL to have a diagnoses of dementia (Alzheimer’s disease) [7]. Given that an individual’s responses on the interRAI CHA are often the main source of information used by the health professional completing the assessment, it is reasonable to expect that the identification of sensory impairment using an interview-based assessment like the interRAI CHA would be more difficult in older adults who have both cognitive and sensory impairments.

The interRAI Community Health Assessment (CHA), and its Deafblind Supplement (DbS) is the only standardized interview instrument for adults (18 years of age or older) that helps first-line health care and social services providers to identify the needs, strengths and challenges for someone with DSL. This instrument is an assessment of overall health and functional abilities that guides an assessor in terms of developing a service plan [11]. The interRAI CHA consists of roughly 150 items, and four supplemental assessments, one of which is the DbS. Responses indicating problems on the two items on the CHA that refer to the person’s functional vision and hearing “trigger” an identification of the person as likely having DSL such that a more comprehensive assessment with the DbS is warranted. The DbS includes an additional 150 items that probe details about the person’s vision and hearing and other issues relevant for an individual with DSL (e.g., age of onset of sensory loss, diagnoses, communication ability, psychosocial well-being) [14]. Based on the information gathered, the assessor then decides how to proceed in terms of referrals (e.g., referral to programs/resources specific to persons with DSL) and implementation of a service plan. Research shows that the interRAI CHA and DbS, and associated scales, have good internal consistency and convergent validity [15,16], as well as some preliminary evidence of acceptable inter-rater reliability [17]. This assessment was created by interRAI (http://www.interrai.org/), a not-for-profit research network of 100 members from 35 countries, who have a mandate to develop and test assessment systems that aim to improve the quality of life and delivery of services for vulnerable populations, including older persons and those with disabilities. The interRAI instruments are based on input from content experts, clinicians and service providers, are used internationally, including being mandated in several regions in Canada and are supported by studies evaluating their psychometric properties [18–20].

While many age-related conditions have been investigated, few studies have examined the assessment of older persons with DSL [26,27]. In particular, the interRAI CHA’s accuracy in detecting single sensory impairment (vision or hearing loss) or DSL has not been examined in comparison to objective measurements of vision and hearing. This study aims is to fill this gap by reporting the sensitivity and specificity of the interRAI CHA for identifying DSL when compared against gold standard performance based measurements of vision and hearing. These measures are important because they assess how well an assessment tool identifies a health condition. Sensitivity evaluates how good is the instrument at identifying true cases; specificity assesses the extent to which the instrument does not mis-identify as true cases respondents that do not have the medical condition.

We administered the interRAI CHA and DbS to 200 older adults (61 years of age or older) known to have VL, HL, or DSL based on performance based measures as recorded in their rehabilitation centre records. Performance based measurements are behavioural tests in which a person’s sensory ability is assessed based on his/her responses to sensory stimuli. Because older adults with sensory loss may also have cognitive impairment, which may limit the accuracy of the screening tool in identifying DSL, this study also reports sensitivity and specificity results for sub-groups of participants categorized as having normal cognition or not based on two cognitive screening measures, the Montreal Cognitive Assessment (MoCA) and Cognitive Performance Scale (CPS). The MoCA was used because it identifies persons with mild cognitive impairment (MCI), and it is frequently used in research and clinical practice. The CPS was used because it is embedded in the interRAI CHA, and classifies persons within several levels of cognitive functioning ranging from none to very severe cognitive impairment (see methods for further details on the MoCA and the CPS).

## Methods

All study procedures were reviewed and approved by the *Centre de recherche interdisciplinaire en réadaptation de Montréal métropolitain* (CRIR-1018-1114). All investigations were performed according to the guidelines of the Declaration of Helsinki and all participants gave informed written consent[28]. This paper followed the STrengthening the Reporting of OBservational studies in Epidemiology (STROBE) guidelines [29]. These guidelines recommend information to be included for the accurate and complete report of an observational study. For instance, these guidelines specify information on the study design, participants, measurements and statistical methods to be included in the methods section.

### Participants

Participants were recruited among older adults that were attending rehabilitation centres because we could obtain their sensory impairment diagnostic information and their performance-based assessments (e.g., acuity). In this way, our recruitment strategy efficiently utilized data already collected in the participants’ clinical records.

Participants met the following four criteria: 1. To be eligible for sensory rehabilitation services as defined by the Quebec Ministry of Health, i.e., a visual acuity in the better eye with best standard correction of 20/60 (6/18) or less, or a visual field diameter of < 60 degrees in the better eye, or hemianopsia (loss of half of the visual field); and/or an unaided pure-tone average decibel hearing loss (dB HL) in the better ear of 35 dB HL or more across 4 frequencies: 0.2, 1, 2, and 4 kHz [30,31]. 2. To have an initial evaluation by a qualified professional at a rehabilitation centre at least 6 months prior to data collection, and receipt of rehabilitation within the past 3 years. This excluded individuals who had recently undergone extensive intake/initial interviews at their respective rehabilitation centres, in order to avoid assessment burden. 3. To be able to communicate verbally in English or French, and to be reachable by phone. 4. To be 61 years old or older. There were no exclusion criteria for participating in this study.

Participants were recruited through the respective programs of three Québec sensory rehabilitation establishments all of which provide service for persons that have VL, HL, or DSL: 1) *CRIR/Centre de réadaptation MAB-Mackay du CIUSSS du Centre-Ouest-de-l’Île-de-Montréal*; 2) *CRIR/Institut Nazareth et Louis-Braille du CISSS de la Montérégie-Centre*; and 3) *CRIR/Institut Raymond-Dewar du CIUSSS du Centre-Sud-de-l'Île-de-Montréal*. Recruitment and data collection took place from August of 2015 to July of 2017. Enrollment procedures are detailed in S1 Text. Supplemental Information Enrollment. The final sample consisted of 200 adults aged 61 and over (61% women, 39% men) with a mean age of 81.3 years. For each participant, records were obtained from the corresponding rehabilitation centre.

### Measures

#### interRAI CHA and DbS

The interRAI CHA consists of roughly 150 items that capture basic demographic information about the person and detailed information across 13 domains (e.g., cognition, social functioning). The assessor enters the answers to the items into a software that generates scores on a series of health index scales, including the Deafblind Severity Index (DbSI). The DbSI is calculated based on two items within the interRAI CHA, one for functional vision and one for hearing, with scores ranging from 0 (no impairment in either sense) to 5 (severe impairment in both senses). Participants with a score greater than 3 (mild/moderate impairment in both senses) are classified as having DSL [11]. For those classified as having DSL, the training manual for the interRAI CHA recommends that the assessor also complete the DbS [11]. The DbS assessment includes an additional 150 items to gather further information across 11 domains considered relevant for the assessment of individuals with DSL (e.g., vision, hearing, communication) [15]. Additional information on the DbS is included in S2 Text. Supplemental Information DbS.

The vast majority of responses options within the interRAI CHA and DbS are closed set; most are scored as yes/no, others are scored on an ordinal scale (typically 0–5, but sometimes up to 8). The assessment items are typically based on a time spanning the previous 3 days; a few items ask about the past 90 days. The assessor completes the interRAI CHA using all available sources of information, including: 1. Reports from respondent and their caregivers and/or health care providers; 2. Respondent’s medical records; and 3. Assessor’s observations regarding the respondent’s functioning during the assessment [11]. Table 1 includes examples of areas assessed by the instrument with corresponding questions and scoring.

**Table 1:** Examples of areas assessed by the interRAI CHA and DbS.

Area Assessed	Question instructions^a^	Scoring
interRAI CHA		
Hearing	Assess the respondent’s hearing ability (with hearing device if used) based on observation and interview of the respondent.	(0) Adequate (1) Minimal difficulty (2) Moderate difficulty (3) Severe difficulty (4) No hearing
Vision	Assess the respondent’s visual ability based on visual tasks and interview of the respondent.	(0) Adequate (1) Minimal difficulty (2) Moderate difficulty (3) Severe difficulty (4) No vision
Cognition	Assess whether the respondent makes decisions about daily living tasks	(0) Independent (1) Modified independence (2) Minimally impaired (3) Moderately impaired (4) Severely impaired (5)No discernable consciousness, coma
DbS		
Vision	Determine whether the respondent’s vision loss is congenital or acquired	(0) 0–2 years (1) 3–18 years (2) 19–64 years (3) 65+ years
Hearing	Determine the person’s ability to respond diverse sounds	(0) Responds without hearing device (1) Responds with hearing device (2) Does not respond

*Note*. CHA = Community Health Assessment; DbS = Deafblind Supplement.

^a^ = table includes a summary of instructions, for full instructions please see [11].

The assessor in the present study was a social worker with over 30 years of direct clinical experience working with adults and older adults that have visual impairment or DSL. She participated in a two-day education session on how to administer the interRAI CHA and DbS based on the information available in the manual produced by interRAI. The assessor secured participants’ written consent, most of whom (61%) chose to be interviewed at their homes and the remaining participants preferred an office appointment at their respective rehabilitation centre (*CRIR/Centre de réadaptation MAB-Mackay du CIUSSS du Centre-Ouest-de-l’Île-de-Montréal* = 38.5%, *CRIR/Institut Raymond-Dewar du CIUSSS du Centre-Sud-de-l'Île-de-Montréal* = 0.5%). Interviews lasted approximately 90 minutes, and were conducted in the participant’s preferred service language, either English or French. Study team members had access to participants’ files from their respective rehabilitation centres. From the files, demographic characteristics (e.g., sex, age) and health information (e.g., vision and/or hearing diagnoses) was obtained. In scoring the assessment items, the assessor considered both the participants’ answers and information from participants’ rehabilitation centre records (when available). The assessor did not have access to the rehabilitation centre records of 70 participants with HL, and had access to only the visual rehabilitation centre records of 62 participants with DSL. Further details regarding data collection are included in S3 Text. Supplemental Information Data collection.

Once the interRAI CHA was completed, responses were entered into a software system using unique identifiers to ensure confidentiality, and the DbSI score was calculated. Because this study defined each participant’s true sensory loss diagnoses based on their medical performance based measurements (rather than their DbSI scores), we chose to complete the DbS on all study participants, regardless of their DbSI score, so that these results were available for the entire sample.

To obtain the assessor’s rating of the participants’ cognitive status, we calculated the Cognitive Performance Scale (CPS), a health index scale [32]. The CPS describes the participant’s cognitive status based on questions and observations regarding short term memory (the respondent is requested to describe a recent event), decision making (whether the respondent actively takes decisions regarding daily living tasks), making self-understood (documenting the respondent’s ability to communicate and engage in social conversation), and ability to eat independently (determining how the respondent eats and drinks). Like all outcomes of the interRAI CHA, the CPS is based on information from multiple sources including the respondent’s self-report and the assessor’s clinical impression. The CPS classifies participants into 7 levels of cognitive performance: intact (0), borderline intact (1), mild impairment (2), moderate (3), moderate/ severe (4), severe (5), and very severe (6). In the CPS, the classification “mild cognitive impairment” refers to the person’s degree of cognitive impairment and it is not equal to the clinical diagnosis of MCI.

#### Montreal Cognitive Assessment (MoCA)

The MoCA, a 10-minute behavioral cognitive screening measure standardized as screening tool for detecting MCI, includes tasks that assess attention, concentration, working memory, short-term memory, visuospatial abilities, executive functions, language, and orientation to time and place (see [33] for further detail). Failing the MoCA indicates that the respondent has mild cognitive difficulties which could be caused by several etiologies, one of which is MCI. Therefore, failing the MoCA does not constitute a diagnosis of MCI, but rather indicates that the respondent should complete the full clinical assessment required to determine an MCI diagnosis. The original version of the MoCA (hereafter referred to as the “full” MoCA) was developed for and validated in adults without controlling for sensory impairment [33]. A version that would not disadvantage persons with vision loss (hereafter “blind” MoCA) was created by eliminating the first 4 items of the scale that require seeing stimuli (i.e., visual trail making, copying a figure, and drawing a clock) and adjusting the cut-off scores for passing accordingly. Compared to the full MoCA, the blind version has higher specificity for detecting normal participants and lower sensitivity for detecting those at risk for MCI [34]. Table 2 lists the tasks included in the MoCA and the corresponding cognitive areas they assess. After data collection had been completed, this study’s authors learned about the MoCA version adapted for individuals with hearing loss [35]. Consequently, this MoCA version was not included in this study’s measurements.

**Table 2:** MoCA tasks and cognitive areas assessed.

Tasks (in presentation order)	Cognitive area assessed by task
1. Alternation task adapted from the Trail Making B task in which a sequence is drawn by connecting symbols printer on paper (excluded in Blind MoCA)	Executive functions
2. Using pen and paper, copy a three-dimensional cube drawing 3. Draw a clock, including all numbers, and the time set to 11:10 (excluded in Blind MoCA)	Visuospatial abilities
4. Name each of three low-familiarity animals (lion, rhinoceros, camel) presented simultaneously as drawings (excluded in Blind MoCA)	Language
5. Two learning trials of a list of 5 spoken nouns with immediate recall	Short-term memory
6. Sets of spoken digits to be recalled in the same (forward) or reverse (backward) order as presented 7. Tap with hand every time the letter A is heard in a list of letters 8. Starting from 100 count by subtracting 7 until told to stop	Attention, concentration, working memory
9. Repetition of two syntactically complex sentences presented orally	Language
10. For 1 minute, tell words starting with a given letter (except proper nouns, numbers, words with the same first letter but different suffix)	Language, executive functions
11. Verbally explain what each of two pair of words have in common	Executive functions
12. Name as many of the 5 nouns learned in task # 5 as recalled	Short term memory
13. Name today’s date, month, year, day of the week, current place and city	Orientation to time and place

*Note*. MoCA = Montreal Cognitive Assessment.

Two participants in the present study did not complete the MoCA. One participant died before he could be assessed, and another participant was not assessed because the assessor felt that the test would have caused the participant undue psychological stress. Participants that were classified by their rehabilitation centres as having a visual impairment (whether VL only or DSL) completed the blind MoCA [33], which has a maximum of 22 points, with a score of 18 points or greater indicating normal cognition [34]. Participants who were not classified as having a visual impairment completed the full MoCA, which has a maximum of 30 points, a score of less than 26 points indicates risk of MCI. The MoCA was typically administered during the interview when the interRAI CHA was completed, with the exception of 5 participants who completed the MoCA at a second session.

#### VL, HL, and DSL assessed with performance based measurements

Study team members had access to participants’ files from their respective rehabilitation centres. From the files, demographic characteristics (e.g., sex, age) and health information (e.g., vision and/or hearing diagnoses) was obtained.

Whether a participant had VL, HL, or DSL based on performance based measurements of hearing and vision was determined using information from rehabilitation centre records, obtained from the participants corresponding rehabilitation centres. In these records, the rehabilitation centre classified the participant as having either VL, HL, or DSL, according to the following performance based measurement criteria:

VL: a visual acuity in the better eye with best standard correction of 20/60 ft (6/18 m) or less, or a visual field diameter of < 60 degrees in the better eye, or hemianopsia (loss of half of the visual field).

HL: an unaided pure-tone average decibel hearing loss (dB HL) in the better ear of 35 dB HL or more across 4 frequencies: 0.2, 1, 2, and 4 kHz.

DSL: criteria for both VL and HL.

In addition, if the participant was classified as having only vision loss by the rehabilitation centre, and he/she also used a hearing aid, the participant was classified as having DSL according to performance based measurements, for the purpose of this study.

### Data analysis

#### Participants’ characteristics

Using both categorical and continuous variables obtained from participants’ rehabilitation centre records, descriptive statistics were calculated to characterize the sample in terms of type of sensory loss (VL only, HL only, DSL), sex, age, hearing (e.g., average decibel hearing loss in the better ear across 4 frequencies: 0.2, 1, 2, and 4 kHz) and vision (e.g., distance visual acuity: ETDRS chart at 4 meters in the better eye with best standard correction). When averages of sensory measurements were calculated, participants were excluded if their rehabilitation centre record lacked the corresponding data. Visual acuity data were missing for 2 participants with DSL. Additionally, 4 participants with VL and 4 of those with DSL had visual acuity levels that could not be expressed in logMAR, and thus were excluded from the calculation of better eye average visual acuity. Contrast sensitivity data were missing for 29 participants with VL and 22 of those with DSL. Right eye horizontal visual field data were missing for 10 participants with VL and 12 of those with DSL. Right eye vertical visual field data were missing for 11 participants with VL, and 12 of those with DSL. Left eye horizontal visual field data were missing for 8 participants with VL, and 12 of those with DSL. Left eye vertical visual field data were missing for 9 participants with VL, and 13 of those with DSL. Average decibel hearing loss data for each ear were missing for 28 participants with DSL and one participant with HL. Data about usage of hearing aid were missing for 16 participants with DSL. Data on cochlear implant usage was missing for 17 participants with DSL. The performance based measurements of vision and hearing were not performed by this study’s team but rather by optometrists and audiologists working with the rehabilitation centres.

The time elapsed from the date of the vision assessment included in the rehabilitation centre records to the completion of the interRAI CHA was on average 10 months, 24 days, and the range was 5 days to 4 years, 7 months, 10 days. Before calculating the average elapsed time for the hearing assessment, data from one participant with HL were considered an outlier and excluded. For this participant, the elapsed time between the hearing examination and the completion of the interRAI CHA was 78 years, 5 months, 5 days, possibly due to the early onset of a HL condition that required no further examination. After excluding this participant, the elapsed time between hearing assessment and interRAI CHA completion was on average 1 year, 8 months, 12 days, range of 14 days to 9 years, 23 days. It should be noted that medical records from rehabilitation centres may not always be complete or up-to-date, given these centres’ focus on functional limitations (e.g., activities of daily living) rather than medical follow-up.

Descriptive statistics were also calculated using data from the interRAI CHA and DbS since these sources provided information that is not typically found in medical assessments included in rehabilitation centre records. For instance, whereas medical assessments contained measurements of the functioning of the eye (e.g., visual acuity), they did not include an assessment of how the participant functioned in vision-related activities (functional vision) [36], which was obtained from the interRAI CHA and DbS. Using data from the interRAI CHA and DbS, the sample was described with regards to functional use of vision and hearing, communication and cognitive performance, and demographic characteristics (e.g., sex, age, education, marital status).

To determine if measurements of visual acuity and contrast sensitivity differed significantly between the VL and DSL groups, independent sample t-tests were calculated. For visual fields, Mann-Whitney tests were conducted (t-test assumptions were not met). To determine if the better ear’s average decibel hearing loss differed significantly between the HL and DSL groups, the Mann-Whitney test was used (t-test assumptions were not met). To investigate associations between group and sex, a chi-square test was conducted. To determine if the sensory impairment groups differed significantly with respect to average age, ANOVA (age as a continuous outcome variable), Post-hoc Tukey and Bonferroni tests were calculated. To investigate if there was an association between sensory impairment group and age (ages grouped into ranges), a chi-square test was calculated. For all analyses, statistical significance was set at p = 0.05. Data were analyzed using SAS^®^ and JASP software.

#### Participants’ cognitive status

Based on the sample’s MoCA scores, participants were classified into two groups: 1. Failed the MoCA (score <26/30 for the full MoCA or <18/22 for the Blind MoCA), 2. Passed the MoCA). The percentage of participants who belonged to each of these two groups was initially calculated for the entire sample and then divided by sensory impairment classification (VL, HL, DSL) as determined by performance based measures. Similar analyses were carried out using the CPS scores as a measure of cognitive status. Participants were grouped into those who had a CPS score of 0 indicating intact cognitive status, and those with scores of 1 or 2, indicating borderline intact cognitive performance or mild degree of impairment. Is important to recall that the CPS classification of “mild cognitive impairment” refers to the degree of the impairment and it is not equal to the clinical diagnosis of MCI.

Researchers may more commonly use the MoCA than the CPS when screening for cognitive status, and may be unfamiliar with the CPS scores and their interpretation. Therefore, the results of these two measurements were compared in order to provide a reference for future analyses. For this purpose, we calculated two statistics: 1. McNemar’s test to determine if there was an association between MoCA and CPS scores. Chi-square was not used since the assumptions were not met in multiple cases; 2. Spearman’s correlation to determine the extent to which the MoCA and CPS scores vary in the same or opposite direction. Spearman’s correlation was used instead of Pearson’s correlation because the CPS is measured on an ordinal scale and assumptions of linearity and homoskedasticity were violated. Because we applied two versions of the MoCA, each with a different criterion score for failing the screening tool (less than 26 points in the Full MoCA; less than 18 points in the Blind MoCA), the interpretation of a given score was not identical across participants that had completed different MoCA versions. For instance, a score of 19 meant passing the MoCA for a participant that had doe the Blind version, whereas it meant failing for a participant that completed the Full version. Therefore, before the Spearman’s correlation was calculated, participants’ scores were expressed as percentage of the maximum possible score according to the MoCA version they completed.

#### Sensitivity and specificity of the interRAI CHA

The sensitivity and specificity of the vision and hearing items on the interRAI CHA were calculated for those with DSL, VL only, or HL only. Table 3 exemplifies this calculation for DSL.

**Table 3:** Sensitivity and specificity calculation for DSL.

	Performance based measure	
InterRAI CHA	DSL	No DSL
DSL	A (true positive)	B (false positive)
No DSL	C (false negative)	D (true negative)
	Sensitivity = $\frac{A}{A+C}$	Specificity = $\frac{D}{D+B}$

*Note*. CHA = Community Health Assessment (CHA); DSL = dual sensory impairment.

Sensitivity was estimated as the ratio of the number of participants detected by the performance based measurements (the gold standard) as having DSL who were *also* identified by the interRAI CHA (cell A), to the number of people classified as having DSL by the performance based measurements (cells A + C). Specificity was estimated as the ratio of the number of people who were identified as *not* having DSL by the performance based measurements and *also* the interRAI CHA (cell D), to the number of people classified as *not* having DSL by the performance based measurements (cells D + B). Sensitivity was therefore defined as the proportion of true DSL cases (e.g., diagnosed as such per performance based measurements) that were correctly identified by the instrument as having DSL and likewise, specificity was defined as the proportion of true *non*-DSL cases that were correctly identified by the instrument as *not* having DSL. Calculations were done including all participants, and also grouped by sex.

#### Sensitivity and specificity divided according to MoCA and CPS result

To determine if sensitivity and specificity were influenced by performance on the MoCA, sensitivity and specificity calculations were redone with participants groups according to whether they passed or failed the MoCA. Lastly, to determine if sensitivity and specificity were influenced by CPS results, the calculations were divided into a CPS score of 0, indicating intact versus a CPS score of 1 or 2, indicating borderline intact cognitive performance or mild degree of impairment. Even though the CPS score can take on values ranging from 0 to 5, there were no participants in this study with a CPS score above 2.

## Results

### 1. Participant characteristics

Based on participants’ rehabilitation centre records, 32.5% (N = 65) of the sample had VL only, 35% (N = 70) had HL only, and 32.5% (N = 65) had DSL. Measurements of visual function did not differ significantly between the VL and DSL groups. In terms of the better eye’s distance visual acuity (logMAR), there was no significant difference between participants with VL (0.88) and DSL (0.91); *t*(118) = -0.49, *p* = 0.63, d = 0.09. Likewise, there were no significant differences between the VL and DSL groups’ average log contrast sensitivity (VL = 1.10, DSL = 1.10, *t*(77) = -0.41, *p* = 0.68) and visual fields (see Table 3 for mean values, Mann-Whitney tests ranging *U* = 0.52–0.72, r_biserial_ ranging 0.04–0.09). A significant difference was found with respect to the better ear’s pure-tone average hearing loss (dB HL). Individuals with HL had significantly higher (worse) pure-tone average hearing loss in the better ear (62.05 dB HL) compared to those with DSL (51.32 dB HL), Mann-Whitney test *U* = 1728.50, *p* = 0.03, r_biserial_ = 0.35.

Table 3 shows the percentage and number of participants according to sex and age for each sensory impairment group. There was no significant association between sex and sensory impairment group, χ^2^ (2, N = 200) = 2.07, p = 0.35. There was a main effect of age, ANOVA *F*(2, 197) = 10.91, MSE = 63.73, *p* < .001, ω^2^ = 0.09. Post-hoc Tukey and Bonferroni tests showed that participants with DSL had a significantly higher mean age (mean = 84.91 years) compared to participants with VL (mean = 80.57 years) and compared to participants with HL (mean = 78.60 years) (p < .05 and .001, respectively). All other comparisons were not significant. Considering the whole sample, most participants were 85 years of age or old (41.5%, N = 83), followed by 75–84 (34%, N = 68), and then 61–74 years (24.5%, N = 49) and these proportions were significantly associated with sensory impairment group, χ^2^ (4, N = 200) = 18.69, p < 0.001. There was a higher proportion of individuals with DSL in the 85+ years age group, compared to those with VL and HL.

Table 4 shows the sample’s characteristics obtained from rehabilitation centre records. Participants’ sensory impairment group classification is based on the performance based measures. Rehabilitation centre records listed all visual loss diagnoses a participant had, Table 1 lists the diagnoses that were the most prevalent in the sample. This table does not include HL diagnoses because they were not tracked in the participants’ rehabilitation records. Participants’ HL diagnoses were obtained from the DbS and are shown in Table 5.

**Table 4:** Characteristics of study participants based on information obtained from rehabilitation centre records.

	Vision loss only (VL) N = 65	Hearing loss only (HL) N = 70	Dual sensory loss (DSL) N = 65
	% (n)^a^		
**Sex**			
Female	60.0 (39)	55.7 (39)	67.7 (44)
Male	40.0 (26)	44.3 (31)	32.3 (21)
**Age**			
Mean (SD)	80.57 (8.08)	78.60 (7.23)	84.91 (8.63)
61–74 years	27.7 (18)	28.6 (20)	16.9 (11)
75–84 years	33.9 (22)	45.7 (32)	21.5 (14)
85+ years	38.5 (25)	25.7 (18)	61.5 (40)
**Hearing**			
Better ear average decibel hearing		62.05 (20.47)	51.32 (15.75)
loss (mean, SD)			
Hearing aid left ear only	N/A	14.3 (10)	7.7 (5)
Hearing aid right ear only		21.4 (15)	9.2 (6)
Hearing aid both ears		58.6 (41)	52.3 (34)
Cochlear implant left ear only		1.4 (1)	0.0
Cochlear implant right ear only		1.4 (1)	0.0
Cochlear implant both ears		1.4 (1)	1.4 (1)
**Vision**			
*Main vision diagnosis*^*b*^			
Age related macular degeneration	55.4 (36)		69.2 (45)
Glaucoma	23.1 (15)		21.5 (14)
Diabetic retinopathy	9.2 (6)		1.5 (1)
Retinitis pigmentosa	7.7 (5)		6.2 (4)
Better eye distance visual acuity	0.88 (0.50)		0.91 (0.40)
(mean, SD)			
*Contrast sensitivity*		N/A	
Log CS (mean, SD)	1.10 (0.40)		1.10 (0.40)
Percent (mean, SD)	13.0 (13.41)		11.0 (9.13)
*Visual field (mean*, *SD)*			
Right eye horizontal	75.01 (53.12)		83.23 (46.80)
Right eye vertical	60.79 (39.66)		64.46 (36.57)
Left eye horizontal	77.19 (47.23)		79.93 (45.67)
Left eye vertical	60.10 (38.12)		63.69 (35.93)

*Note*. SD = standard deviation; logMAR = logarithm of the minimum angle of resolution; Log CS = logarithm of contrast sensitivity.

^a^ Percentage and number of participants are calculated separately for each sensory impairment group.

^b^ Only the top 4 most prevalent visual diagnoses are reported.

**Table 5:** Sensitivity and specificity results for Vision Loss (VL).

	Performance based measure	
InterRAI CHA	VL	No VL
VL	65	1
No VL	0	134
	Sensitivity: 100%	Specificity: 99.3%

*Note*. CHA = Community Health Assessment (CHA); DSL = dual sensory loss.

Based on the interRAI CHA and DbS, most individuals with VI (90.8%) and with DSL (80%) were classified as having moderate vision difficulty. Most participants with HL (57.1%) and DSL (76.9%) were classified as having minimal difficulty with hearing. A higher percentage of participants with HL (35.7%) were classified as having moderate hearing difficulty, compared to those with DSL (18.5%).

Regarding highest educational level, there was a significant association with sensory impairment group, Fisher p = 0.04. A higher percentage (48.6%) of participants with HL had post-secondary education, followed by DSL (36.9%), and then VL (32.3%). A higher percentage (32.3%) of participants with VL had high school or trade school, compared to DSL (24.6%), and HL (18.6%). With respect to residential status, there was a significant association with sensory impairment group, Fisher p = 0.03. In all sensory impairment groups, most participants (75.4% to 88.6%) lived in a private home or apartment or in a rented room. A lower percentage (7.5%) of participants with HL lived in assisted-living or semi-independent living residences, compared to VL (20%), and DSL (23.1%). See S1 Table. interRAI CHA DbS data for the sample’s characteristics based on the interRAI CHA and DbS.

#### Participants’ cognitive status

Overall, based on both MoCA and CPS results, most participants were not experiencing difficulties in their cognitive functioning. With respect to the MoCA, 58.6% (N = 116) passed the test. There was no association between MoCA result (i.e., Pass/Fail) and sensory impairment group, Fisher p = 0.86. Among the participants who passed the MoCA, 33.6% (N = 39) had VL, 33.6% (N = 39) had DSL, and 32.8% (N = 38) had HL. Of the participants that failed the MoCA, 37.8% had HL (N = 31), 31.7% had DSL (N = 26), and 30.5% had VL (N = 25). Within each sensory impairment group, more than 50% of participants passed the MoCA. Table 3 shows the percentages of participants that passed and failed the MoCA, divided by sensory impairment group.

Based on the CPS, all participants scored zero, one or two on the scale. Furthermore, in all sensory impairment groups most participants (over 91%) were assessed as cognitively intact (CPS = 0). Therefore, the sample had a very limited distribution of cognitive impairment with only 4 out of the 200 participants showing mild cognitive impairment (recall that according to the CPS, “mild cognitive impairment” refers to the degree of the impairment and it is not equal to the clinical diagnosis of MCI). There was no association between CPS result and sensory impairment group, Fisher p = 0.87. In each sensory impairment group the vast majority of participants were categorized as cognitively intact (VL = 91.4%, HL = 95.6%, DSL = 94.5%), followed by borderline intact (VL = 5.2%, HL = 2.9%, DSL = 4.1%), and then mild impairment (VL = 3.5%, HL = 1.5%, DSL = 1.4%).

The proportion of individuals who passed the MoCA was significantly different than the proportion of individuals who obtained a CPS of zero, χ^2^_McNemar_ (1, N = 198) = 88.66, p < 0.0001, indicating that these two cognitive measures may not equally determine normal cognitive functioning. When this analysis was done separately for each sensory impairment group, results were significant for VL, χ^2^_McNemar_ (1, N = 64) = 23.27, p < 0.0001; HL, χ^2^_McNemar_ (1, N = 69) = 31.41, p < 0.0001; and DSL, χ^2^_McNemar_ (1, N = 65) = 35.10, p < 0.0001. Whereas only 8% of the sample obtained a CPS score indicating some degree of cognitive impairment, 41.4% of the sample failed the MoCA. Most participants who passed the MoCA (97.4%), but also most participants who failed it (89.0%), were considered to be cognitively intact based on the CPS. In nine cases (11%), the CPS identified participants as having some degree of cognitive impairment which was in line with a risk of MCI based on the MoCA. Only participants that failed the MoCA were classified as having mild impairment by the CPS (4.9%).

Results of the Spearman correlation indicated that there was a statistically significant negative correlation between the CPS and MoCA scores, *r*_Spearman_ (196) = -0.26, p < 0.001. Because higher CPS scores indicate worse cognitive functioning whereas higher MoCA scores indicate better cognitive functioning, this correlation indicates that the CPS and the MoCA scores vary in the same direction (i.e., are positively correlated).

### 2. Sensitivity and specificity of the interRAI CHA for identifying VL, HL, and DSL

Overall, the sensitivity and specificity values for the interRAI CHA for detecting VL, HL, and DSL were high. Sensitivity was highest for identifying VL only (100%), followed by HL (97.1%) and then DSL (96.9%). Two cases that were classified as having DSL based on the rehabilitation centre records were identified as having only VL by the instrument. These two participants did not report difficulties with hearing when responding to the interRAI CHA question on hearing, and therefore did not meet the DbSI criteria to be identified as having DSL. Specificity was highest for identifying VL (99.3%), followed by HL (93.1%) and then DSL (92.6%). There were 10 cases of disagreement; their true diagnosis was single sensory impairment but the interRAI CHA classified them as having DSL. In nine of these cases, participants reported having minimal hearing difficulty and thus were classified as having DSL by the interRAI CHA whereas the rehabilitation centre record identified them as having VL only. In the remaining case, the participant reported having minimal vision difficulty and was classified as having DSL by the instrument whereas the rehabilitation centre record identified them as having HL only. Tables 5, 6, and 7 display the sensitivity and specificity results for VL, HL, and DSL, respectively. Sensitivity and specificity results were virtually identical when males and females were compared (S2 Table. Results by sex).

**Table 6:** Sensitivity and specificity results for Hearing Loss (HL).

	Performance based measure	
InterRAI CHA	HL	No HL
HL	68	9
No HL	2	121
	Sensitivity: 97.1%	Specificity: 93.1%

*Note*. CHA = Community Health Assessment (CHA); DSL = dual sensory loss.

**Table 7:** Sensitivity and specificity results for Dual Sensory loss (DSL).

	Performance based measure	
InterRAI CHA	DSL	No DSL
DSL	63	10
No DSL	2	125
	Sensitivity: 96.9%	Specificity: 92.6%

*Note*. CHA = Community Health Assessment (CHA); DSL = dual sensory loss.

### 3. Sensitivity and specificity according to cognitive status

Sensitivity was 100% for VL only; therefore, we were not able to further explore the potential effect of cognitive status on the interRAI CHA’s sensitivity for detecting VL only in this sample. Based on the MoCA results, the sensitivity of the interRAI CHA to detect HL and DSL was virtually identical among participants that failed the test (96.8% and 96.2% respectively), compared to participants that passed it (97.4% for each sensory impairment group). Specificity for identifying HL and DSL was somewhat lower for participants that failed the MoCA (90.2% and 91.1% respectively), versus those that passed it (96.2% and 94.8% respectively). In contrast, specificity for detecting VL was slightly higher in participants that failed the MoCA (100%), compared with those who passed it (98.7%). This was due to one participant who passed the MoCA, was *not* classified as having VL by the rehabilitation centre record, and was reported as having vision difficulties on the interRAI CHA. Table 8 summarizes the sensitivity and specificity results according to cognitive status for all sensory impairment groups. See S3 Table. Results by cognitive status for tables that display the sensitivity and specificity according to cognitive status separately per sensory impairment group.

**Table 8:** The sensitivity and specificity of the interRAI CHA based on the MoCA score and risk of MCI.

Sensory	Sensitivity		Specificity	
Group	*No* Risk of MCI	Risk of MCI	*No* Risk of MCI	Risk of MCI
*VL*	100%	100%	98.7%	98.5%
*HL*	97.4%	96.8%	96.2%	90.2%
*DSL*	97.4%	96.2%	94.8%	91.1%

*Note*. VL = vision loss; HL = Hearing loss; DSL = dual sensory loss; MCI = mild cognitive impairment.

Based on the CPS results, the sensitivity of the interRAI CHA for detecting HL and DSL was somewhat higher in participants who were categorized as being borderline intact (CPS = 1) or having mild cognitive impairment (CPS = 2) at 100% sensitivity, versus those deemed cognitively intact (CPS = 0) with sensitivities at 97.0% and 96.8%, respectively. Specificity for detecting VL was virtually identical among participants evaluated as borderline intact or as having mild cognitive impairment (specificity of 100%), compared to those assessed as cognitively intact (specificity of 99.2%). In contrast, specificity for detecting HL and DSL was better for cognitively intact participants (94.2% and 93.6%, respectively), versus those assessed as borderline intact or as having mild cognitive impairment (77.8% and 80%, respectively). Table 9 summarizes the sensitivity and specificity results based on the CPS score.

**Table 9:** The sensitivity and specificity of the interRAI CHA based on the CPS score.

Sensory Group	Sensitivity		Specificity	
	Cognitively Intact	Borderline Intact/MCI	Cognitively Intact	Borderline Intact/MCI
*VL*	100%	100%	99.2%	100%
*HL*	97.0%	100%	94.2%	77.8%
*DSL*	96.8%	100%	93.6%	80%

*Note*. VL = vision loss; HL = Hearing loss; DSL = dual sensory loss; MCI = mild cognitive impairment.

## Discussion

The interRAI CHA showed very strong sensitivity and specificity in identifying sensory impairment in this sample of older adults receiving sensory rehabilitation services. Regardless of sensory impairment level, these values were over 90% in all cases. To our knowledge, this is the first study to evaluate the ability of the interRAI CHA to correctly detect sensory impairments. The conditions under which the interRAI CHA was completed in this study (i.e., conducted by an experienced social worker, to adults receiving sensory rehabilitation, with access to some of the respondents’ rehabilitation centre records) do not represent all possible conditions under which first care health providers may complete the interRAI CHA. Our results support further research under different assessment conditions (i.e., novice assessor, no access to rehabilitation centre records) to determine whether our high sensitivity and specificity results can be generalized to other contexts.

In reviewing the discrepancies between the interRAI CHA and the performance based measurements, it is important to consider that the interRAI CHA identifies vision and hearing difficulty in respondents using sensory aids. This makes sense from a service referral and plan perspective because in this way the assessment does not conclude that there is a sensory problem that warrants rehabilitation services, when respondents are functioning well with their current rehabilitation tools and strategies. The analysis of the discrepancies in categorizations of sensory loss between the interRAI CHA and the performance based measurements indicates that two respondents that were classified as having HL per performance-based measurements, did not self-report having hearing difficulties when completing the interRAI CHA. These discrepancies may result from the different ways the interRAI CHA and performance based assessments classify HL. Whereas the interRAI CHA asks for an evaluation of the respondent’s hearing abilities while using their sensory aids/adaptations, performance based assessments identify HL in the absence of hearing aids. Consequently, the interRAI may not classify persons who have HL as having HL according to performance based measurements administered without assistive devices, but who experience no hearing difficulty when using sensory aids/adaptations (e.g., false negative). This potential cause of false negatives is not present for the identification of VL since performance based measurements diagnose VL with the person using vision aids. In line with this, our results showed no VL false negatives.

Another factor that may affect the interRAI CHA’s sensitivity relates to the times when the only available information to the assessor is the respondent’s answers. Evidence shows only moderate correspondence between prevalence estimates of vision or hearing loss based older adults’ self-reports, and estimates based on performance based measures [37]. The evidence on this topic suggests that older adults under-report vision or hearing impairment, which undermines the sensitivity of any interview-based assessment for detecting vision and/or hearing loss.

With respect to specificity, the interRAI CHA avoids false positives (e.g., erroneously identifying a sensory impairment) of VL more successfully than of HL and DSL. In all cases, false positives involved participants whom the interRAI CHA assessed as having “minimal difficulty” in a sensory area which was not deemed to be impaired based on the performance based measurements. “Minimal difficulty” refers to experiencing difficulties “in some environments (e.g., when the other person speaks softly)”. It is possible that what the interRAI CHA considers as the minimal level of sensory difficulty is below the objective measurement’s threshold for diagnosing a sensory impairment. Another possibility is that because the interRAI CHA is conducted in uncontrolled acoustical conditions and background noise, respondents may experience hearing difficulties that they do not endure during performance based hearing tests. Alternatively, considering the time elapsed from the vision or hearing examination included in the rehabilitation centre record to the completion of the interRAI CHA (10 months, 24 days and 1 year, 8 months, 12 days, respectively), it is possible that the interRAI CHA is identifying a currently “true” sensory impairment that was not present when the last performance based measurement was taken, and thus is not included in the rehabilitation centre record.

In absolute terms, the interRAI’s sensitivity was higher than specificity, indicating that the instrument performs better at detecting true cases of VL, HL, and DSL than at avoiding false positives. These results are in line with the interRAI CHA’s objective of identifying the most salient health issues in order for the assessor to then determine what additional referral, assessments, and services can benefit the respondent’s health. Given these findings, it would be important for the interRAI CHA assessor to have a strategy to avoid false positives. If the assessor has access to medical records, s/he can identify respondents who report a sensory impairment, yet do not have such diagnosis in their medical records. If the sensory issue in question has not being examined by a medical professional, the service plan should include a recommendation for such an evaluation in order to determine if the respondent truly has a sensory impairment according to performance based measurements. If the sense had been evaluated by a medical professional but found within normal range, it would be important to note how recent the evaluation was completed. The older the evaluation, the more reason to include in the respondent’s service plan as a referral to update the sensory evaluation.

Sensitivity to detect HL and DSL was virtually identical among participants that failed the MoCA and those that failed it. In absolute terms, sensitivity for HL was 0.6% lower and for DSL 1.2% lower in participants that failed the MoCA, compared to those not that passed it. Specificity was somewhat lower in participants that failed the MoCA, versus those that passed it. The lowest specificity observed was among individuals that failed the MoCA who had either HL or DSL. In both cases, the specificity was roughly 8% lower (in absolute terms) compared to those with VL only. Overall, these findings suggest that the accuracy with which the interRAI CHA identifies sensory impairment is high even in participants that fail the MoCA (ranging between specificity of 90.2% and sensitivity of 100%). Nonetheless, avoiding false positives can be somewhat more difficult in respondents that, in addition to sensory impairment, have mild cognitive difficulties as defined by the fact that they failed the MoCA. If an assessor knows from a respondent’s medical record that the respondent has failed the MoCA, s/he should be especially careful to utilize all available sources of information, besides the respondent’s responses, when assessing sensory functioning. According to the interRAI CHA guidelines, an assessor must seek out and use sources of information other than the respondent’s answers. This is particularly critical if the assessor suspects that the respondent had difficulties understanding questions and/or expressing responses.

Sensitivity for all sensory impairment groups and specificity for VL were high (over 96%) in participants classified by the CPS as cognitively intact, as well as those deemed borderline intact or mildly cognitively impaired. In comparison, specificity for HL and DSL was higher for cognitively intact participants versus those assessed as borderline intact or as having mild cognitive impairment. Given the very limited distribution of CPS scores in our sample, we highlight that our findings cannot be generalized beyond the range of CPS scores available within our sample.

This study’s results should be evaluated taking into consideration several limitations. One, we considered that the true sensory impairment diagnosis of a respondent was determined by performance based behavioural measurements obtained from rehabilitation centre records. However, the rehabilitation centre record may not be up to date with regard to the participant’s sensory status and may not correspond to the true sensory state at the time of participation. Although we recognize this as a study limitation, our high sensitivity and specificity results suggest that the time elapsed between the performance based measurements and the interRAI CHA did not introduce a significantly bias to this study’s results. Even if the rehabilitation centre record is up to date, a person’s sensory functioning under the very specific sensory conditions under which performance based measurements are conducted, may not reflect the person’s sensory functioning in everyday situations [38]. Furthermore, the criteria used by the rehabilitation centres to determine if a respondent has a sensory impairment may exclude older adults that present with milder forms of sensory impairment. Two, several characteristics of our sample limit the generalizability of our findings. All participants were receiving sensory rehabilitation services, which likely made them more self-aware of their sensory status and better able to respond to the sensory items on the interRAI CHA, compared to persons with sensory loss who are not receiving rehabilitation services. The fact that all participants were able to get themselves to their rehabilitation centres, and to participate in their activities, suggests a level of functionality that may be greater than in persons who do not attend rehabilitation centres. According to the CPS results, our sample had a very limited distribution of scores (i.e., only *mild* cognitive impairment was represented), with most participants being classified as cognitively intact or borderline intact. Three, following the interRAI CHA instructions, this study assessor used all available sources of information to complete the instrument. In some cases, these included the respondent’s rehabilitation centre records, if available. This ability to access rehabilitation centre records for some respondents but not others is a common context in which the instrument is completed by first-line health care providers. However, it can also happen that assessors do not have access to any rehabilitation centre records, a situation that is not investigated in this study.

Future research is needed to investigate remaining questions. Because in our study sensitivity was 100% for VI participants, further studies are needed to further investigate the effect of risk of MCI for detecting VL only. This study assessed participants with VL, but not participants with HL, with a MoCA version adapted to their sensory impairment. Future research should investigate the effect of failing the MoCA for detecting HL and DSL, using the now available MoCA version adapted for respondents with HL [35]. Future research should expand the generalizability of findings by studying participants with a wider array of cognitive impairment levels and who are not receiving rehabilitation services.

In summary, the interRAI CHA detects VL only, HL only, and DSL with strong sensitivity and specificity, compared to performance based measurements of vision and hearing. The instrument shows high accuracy even in participants who have cognitive difficulties. Still, assessors should be mindful that identifying sensory impairment using the interRAI CHA is somewhat more difficult in respondents who in addition to sensory impairment, have cognitive difficulties. These findings support further research to investigate whether our high sensitivity and specificity findings generalize to the diverse assessment conditions in which first-line health care providers may use the interRAI CHA for the detection of sensory impairments in older adults, including those with cognitive difficulties.

## Supporting information

S1 Text Supplemental information enrollment. (DOCX)

S2 Text Supplemental information DbS. (DOCX)

S3 Text Supplemental information data collection. (DOCX)

S1 Table interRAI CHA DbS data. (DOCX)

S2 Table Results by sex. (DOCX)

S3 Table Results per cognitive status. (DOCX)
