# Supplementary material for: Detection of vision and /or hearing loss using the interRAI Community Health Assessment aligns well with common behavioral vision/hearing measurements
Source: PLoS One. 2019 Oct 3;14(10):e0223123. doi: 10.1371/journal.pone.0223123 (PMC6776414; doi:10.1371/journal.pone.0223123)
Supplement: S1 Text — (DOCX) [file pone.0223123.s001.docx]

**S1 Text. Supplemental Information Enrollment.**

**Enrollment procedures**

Among the clients of the *CRIR/Centre de réadaptation MAB-Mackay du CIUSSS du Centre-Ouest-de-l’Île-de-Montréal* (MMRC) are persons with visual loss that participate in the Day Centre, 60% of whom have compromised hearing. Some of this study’s participants, who attended an an inter-establishment program (programme surdicécité) of the *CRIR/Institut Nazareth et Louis-Braille du CISSS de la Montérégie-Centre* (INLB) and the *CRIR/Institut Raymond-Dewar du CIUSSS du Centre-Sud-de-l'Île-de-Montréal* (IRD), were registered in both of those centres. Participants were recruited by staff from each of the three rehabilitation centres. The staff provided eligible participants with information concerning the nature and voluntary participation in the study and gave them an opportunity to ask questions. After eligible participants consented verbally, their names were forwarded to this study’s assessor (CM), who scheduled this study’s interview. After receiving more details about the study protocol, 21 (INLB = 10, IRD = 6, MMRC = 5) of the 221 persons who had initially agreed to participate, cancelled their participation in the study before data collection began, mentioning reasons related to health issues, as well as limited energy.
