# Supplementary material for: Detection of vision and /or hearing loss using the interRAI Community Health Assessment aligns well with common behavioral vision/hearing measurements
Source: PLoS One. 2019 Oct 3;14(10):e0223123. doi: 10.1371/journal.pone.0223123 (PMC6776414; doi:10.1371/journal.pone.0223123)
Supplement: S2 Text — (DOCX) [file pone.0223123.s002.docx]

**S2 Text. Supplemental Information DbS.**

**Deaf Blind Supplement (DbS)**

Among the 11 domains assessed with the DbS are: vision and hearing (e.g., age of onset of vision/hearing loss, diagnoses, visual acuity and field diameter, alerting to sounds, devices used), communication (e.g., communication modes used, ability to communicate with family members), mood and behaviour, level and type of informal support from friends/family, psychosocial well-being, and orientation and mobility/O&M (e.g., ability to move about in both familiar and unfamiliar environments). All of the responses to items within the DbS are closed-ended; most are scored as yes/no, others are scored on an ordinal scale (typically 0-5, but sometimes up to 8). The typical time frame for assessment is the previous 3 days; a few items ask about the past 90 days. Further information is available in the assessment’s manual [1], which is available for purchase from interRAI (<http://www.interrai.org/instruments/>).

**References**

1. Morris J, Berg K, Bjorkgren M, Declercq A, Finne-Soveri H, Fries B, et al. interRAI community health (CHA) assessment form and user’s manual. Version 9.1 (Canadian Edition edition). Washington, D.C.; 2010.
