# Supplementary material for: Detection of vision and /or hearing loss using the interRAI Community Health Assessment aligns well with common behavioral vision/hearing measurements
Source: PLoS One. 2019 Oct 3;14(10):e0223123. doi: 10.1371/journal.pone.0223123 (PMC6776414; doi:10.1371/journal.pone.0223123)
Supplement: S3 Text — (DOCX) [file pone.0223123.s003.docx]

**S3 Text. Supplemental Information Data collection.**

**Data collection**

The education session on how to administer the interRAI CHA and DbS that thi study’s assessor attended included instructions for obtaining information from secondary sources, how to enter the data into the software system, and how to interpret information generated by the interRAI CHA software (e.g., scores on the health index scales). While this study asesor’s had no prior experience administering the interRAI assessments, she had extensive interviewing skills, clinical experience and knowledge in the field of sensory rehabilitation.

The assessor provided participants with a description and basic guidelines concerning the study assessment process that included the topics to be covered, the interview format, and the respondents’ ability to ask clarifying questions and take breaks. Participants chose the location where the interview was conducted. At the interview, participants were asked if they understood or had questions before proceeding. To meet the participant’s individualized needs, the assessor used effective communication strategies, in surroundings suitable for clear two-way communication [1]. To facilitate communication, a personal amplification device (Pocket Talker®), was made available to participants who required an assistive listening device. Similarly, a large print version of the consent form was available, as needed.

**References**

1. Tye-Murray N. Foundations of aural rehabilitation. Children, adults and their family members. 4th ed. Stamford: Cengage Learning; 2015.
