# Supplementary material for: Detection of vision and /or hearing loss using the interRAI Community Health Assessment aligns well with common behavioral vision/hearing measurements
Source: PLoS One. 2019 Oct 3;14(10):e0223123. doi: 10.1371/journal.pone.0223123 (PMC6776414; doi:10.1371/journal.pone.0223123)
Supplement: S1 Table — (DOCX) [file pone.0223123.s004.docx]

**S1 Table. interRAICHA DbS data.**

The table below uses the rehabilitation centre records to classify participants as having VL, HL or DSI. This classification was not always in agreement with the way the interRAI CHA determined participants’ sensory impairment group membership. Consequently, in the table there are cases where participants’ responses do not match their sensory impairment group classification. For instance, age at hearing loss onset is reported for four participants in the VL only group.

**Sample’s characteristics obtained from interRAI CHA and DbS**

|  | **Vision loss only (VL)**  **N = 65** | **Hearing loss only (HL)**  **N = 70** | **Dual sensory impairment (DSI)**  **N = 65** |
| --- | --- | --- | --- |
|  | % (N)^a^ | | |
| Hearing |  |  |  |
| *Main hearing diagnosis^b^* |  |  |  |
| Presbycusis | 1.5 (1) | 72.9 (51) | 72.3 (47) |
| Tinnitus or head/ear noise | 0.0 | 10.0 (7) | 0.0 |
| Meniere's disease | 0.0 | 4.3 (3) | 1.5 (1) |
| *Hearing (using hearing device if applicable)* |  |  |  |
| Adequate | 86.2 (56) | 0.0 | 0.0 |
| Minimal difficulty | 13.9 (9) | 57.1 (40) | 76.9 (50) |
| Moderate difficulty | 0.0 | 35.7 (25) | 18.5 (12) |
| Severe difficulty | 0.0 | 7.1 (5) | 1.5 (1) |
| Vision |  |  |  |
| *Vision (using vision device if applicable)* |  |  |  |
| Adequate | 4.6 (3) | 98.6 (69) | 0.0 |
| Minimal difficulty | 90.8 (59) | 1.4 (1) | 12.3 (8) |
| Moderate difficulty | 0.0 | 0.0 | 80.0 (52) |
| Severe difficulty | 3.1 (2) | 0.0 | 3.1 (2) |
| No vision | 1.5 (1) | 0.0 | 4.6 (3) |
| Demographic information |  |  |  |
| *Education* |  |  |  |
| Less than high school | 20.0 (13) | 8.6 (6) | 24.6 (16) |
| Some high school | 15.4 (10) | 24.3 (17) | 13.9 (9) |
| High school or trade school | 32.3 (21) | 18.6 (13) | 24.6 (16) |
| Post-secondary | 32.3 (21) | 48.6 (34) | 36.9 (24) |
| *Marital status* |  |  |  |
| Never married | 10.1 (7) | 5.7 (4) | 6.2 (4) |
| Married/partner/significant other | 40.0 (26) | 42.9 (30) | 29.2 (19) |
| Widowed/separated/divorced | 49.2 (32) | 51.4 (36) | 64.6 (42) |
| *Primary language* |  |  |  |
| English | 69.2 (45) | 67.1 (47) | 58.5 (38) |
| French | 30.8 (20) | 32.9 (23) | 41.5 (27) |
| *Residential Status* |  |  |  |
| Private home/apartment/rented room | 78.5 (51) | 88.6 (62) | 75.4 (49) |
| Assisted living or semi-independent living | 20.0 (13) | 7.1 (5) | 23.1 (15) |
| Long-term care facility (nursing home) | 0.0 | 0.0 | 1.54 (1) |
| Correctional facility | 0.0 | 1.4 (1) | 0.0 |
| Other | 1.5 (1) | 2.9 (2) | 0.0 |
| *Living with* |  |  |  |
| Alone | 52.3 (34) | 44.3 (31) | 58.5 (38) |
| With spouse/partner only | 36.9 (24) | 44.3 (31) | 30.8 (20) |
| With spouse/partner and other(s) | 3.1 (2) | 7.1 (5) | 6.2 (4) |
| With child (no spouse/partner) | 3.1 (2) | 7.1 (5) | 6.2 (4) |
| With sibling(s) | 0.0 | 0.0 | 3.1 (2) |
| With nonrelative(s) | 4.6 (3) | 2.9 (2) | 0.0 |

^a^ Percentage and number of participants are calculated separately for each sensory impairment group.

^b^ Only the top 3 most prevalent visual diagnoses are reported.
