# Supplementary material for: Detection of vision and /or hearing loss using the interRAI Community Health Assessment aligns well with common behavioral vision/hearing measurements
Source: PLoS One. 2019 Oct 3;14(10):e0223123. doi: 10.1371/journal.pone.0223123 (PMC6776414; doi:10.1371/journal.pone.0223123)
Supplement: S2 Table — (DOCX) [file pone.0223123.s005.docx]

**S2 Table. Results by sex.**

|  | **Sensitivity** | | **Specificity** | |
| --- | --- | --- | --- | --- |
| **Sensory Group** | Female | Male | Female | Male |
| *VL* | 100% | 100% | 99.4% | 100% |
| *HL* | 97.4% | 96.8% | 96.9% | 97.6% |
| *DSL* | 97.7% | 95.2% | 96.2% | 97.8% |

*Note.* VL = vision loss; HL = Hearing loss; DSL = dual sensory loss,

Female N = 122, Male N = 78.
