# Supplementary material for: Detection of vision and /or hearing loss using the interRAI Community Health Assessment aligns well with common behavioral vision/hearing measurements
Source: PLoS One. 2019 Oct 3;14(10):e0223123. doi: 10.1371/journal.pone.0223123 (PMC6776414; doi:10.1371/journal.pone.0223123)
Supplement: S3 Table — (DOCX) [file pone.0223123.s006.docx]

**S3 Tables. Results per cognitive status.**

**Sensitivity and specificity results for Vision Loss (VL), divided by MoCA result (pass or fail)**

|  | Performance based measure | | | |
| --- | --- | --- | --- | --- |
|  | Passed the MoCA | | Failed the MoCA | |
| InterRAI CHA | VL | No VL | VL | No VL |
| VL | 39 | 1 | 25 | 0 |
| No VL | 0 | 76 | 0 | 57 |
|  | Sensitivity: 100% | Specificity: 98.7% | Sensitivity: 100% | Specificity: 100% |

Note. CHA = Community Health Assessment (CHA).

**Sensitivity and specificity results for Hearing Loss (VL), divided by MoCA result (pass or fail)**

|  | Performance based measure | | | |
| --- | --- | --- | --- | --- |
|  | Passed the MoCA | | Failed the MoCA | |
| InterRAI CHA | HL | No HL | InterRAI CHA | HL |
| HL | 37 | 3 | HL | 30 |
| No HL | 1 | 75 | No HL | 1 |
|  | Sensitivity: 97.4% | Specificity: 96.2% | Sensitivity: 96.8% | Specificity: 90.2% |

Note. CHA = Community Health Assessment (CHA).

**Sensitivity and specificity results for Dual Sensory Loss (DSL), divided by MoCA result (pass or fail)**

|  | Performance based measure | | | |
| --- | --- | --- | --- | --- |
|  | Passed the MoCA | | Failed the MoCA | |
| InterRAI CHA | DSL | No DSL | InterRAI CHA | HL |
| DSL | 38 | 4 | DSL | No DSL |
| No DSL | 1 | 73 | 25 | 5 |
|  | Sensitivity: 97.4% | Specificity: 94.8% | Sensitivity: 96.2% | Specificity: 91.1% |

Note. CHA = Community Health Assessment (CHA).
